# TGF beta inhibits HGF, FGF7, and FGF10 expression in normal and IPF lung fibroblasts

**DOI:** 10.14814/phy2.13794

**Published:** 2018-08-28

**Authors:** Kelly A. Correll, Karen E. Edeen, Elizabeth F. Redente, Rachel L. Zemans, Benjamin L. Edelman, Thomas Danhorn, Douglas Curran‐Everett, Amanda Mikels‐Vigdal, Robert J. Mason

**Affiliations:** ^1^ National Jewish Health Denver Colorado; ^2^ Division of Pulmonary and Critical Care Medicine Department of Medicine University of Michigan Ann Arbor Michigan; ^3^ Gilead Sciences Foster City California

**Keywords:** Idiopathic pulmonary fibrosis, KGF, pulmonary fibrosis

## Abstract

TGF beta is a multifunctional cytokine that is important in the pathogenesis of pulmonary fibrosis. The ability of TGF beta to stimulate smooth muscle actin and extracellular matrix gene expression in fibroblasts is well established. In this report, we evaluated the effect of TGF beta on the expression of HGF, FGF7 (KGF), and FGF10, important growth and survival factors for the alveolar epithelium. These growth factors are important for maintaining type II cells and for restoration of the epithelium after lung injury. Under conditions of normal serum supplementation or serum withdrawal TGF beta inhibited fibroblast expression of HGF, FGF7, and FGF10. We confirmed these observations with genome wide RNA sequencing of the response of control and IPF fibroblasts to TGF beta. In general, gene expression in IPF fibroblasts was similar to control fibroblasts. Reduced expression of HGF, FGF7, and FGF10 is another means whereby TGF beta impairs epithelial healing and promotes fibrosis after lung injury.

## Introduction

Idiopathic pulmonary fibrosis (IPF) is a devastating disease whose pathogenesis is not fully understood and has nearly a 50% 3‐year mortality rate (Ley et al. 2011; Nalysnyk et al. 2012). The pathogenesis is thought to involve epithelial injury, aberrant repair, and subsequent fibrosis (Wuyts et al. 2013). TGF beta is an important profibrotic cytokine in the development of pulmonary fibrosis (Sime et al. 1997; Leask and Abraham 2004; Fernandez and Eickelberg 2012). Most reports on TGF beta have focused on matrix production by fibroblasts and surfactant production by alveolar type II cells. In fibroblasts, TGF beta increases ACTA2 (smooth muscle actin) expression, promotes fibroblast gel contraction, and stimulates collagen and extracellular matrix production. TGF beta delivered by an adenoviral vector has been shown to produce extensive pulmonary fibrosis in experimental animals (Sime et al. 1997). The alveolar epithelium is also a target for TGF beta. TGF beta inhibits keratinocyte growth factor (KGF, FGF7) induced type II cell proliferation (Zhang et al. 2004), and inhibition of type II cell proliferation would be expected to contribute to the fibrotic response based on the classic studies of Witschi and Adamson (Adamson et al. 1988, 1990; Witschi 1990; Uhal and Nguyen 2013). TGF beta also reduces the expression of SP‐A, SP‐B, and SP‐C in human type II cells and fetal human lung and adenoviral delivered TGF beta decreases SP‐B and SP‐C in mice (Beers et al. 1998; Lopez‐Rodriguez et al. 2016; Correll et al. 2018). Reduction in surfactant would contribute to the work of breathing, elastic recoil, appositional atelectasis, and hypoxia in pulmonary fibrosis (Lutz et al. 2015).

Fibroblasts secrete important growth factors for the alveolar epithelium that limit or prevent fibrosis and promote the differentiation and maintenance of the alveolar epithelium. These include hepatocyte growth factor (HGF), fibroblast growth factor 7 (FGF7, keratinocyte growth factor, KGF), and FGF10. HGF has been shown to accelerate the closure of human alveolar epithelial cell scratch wounds in vitro (Ito et al. 2014). HGF given as a protein or electroporated as a plasmid reduces bleomycin induced fibrosis in rats and mice (Dohi et al. 2000; Gazdhar et al. 2013). FGF7 markedly stimulates proliferation of alveolar type II cells and improves their differentiated function in vitro (Panos et al. 1993; Zhang et al. 2004). FGF7 also ameliorates lung injury due to acid instillation and prevents fibrosis due to instillation of bleomycin (Yano et al. 1996; Deterding et al. 1997; Guo et al. 1998; Sugahara et al. 1998). FGF10 is expressed in lipofibroblasts and is stated to provide a niche for type II cells (El Agha et al. 2014; Volckaert and De Langhe 2014; Chao et al. 2016).

Since TGF beta is critical in the pathogenesis of IPF and HGF, FGF7, and FGF10 are important in alveolar epithelial repair, we evaluated the effect of TGF beta on regulating HGF, FGF7, and FGF10 in both control and IPF human fibroblasts. Since many of the studies on the effects of TGF beta on pulmonary fibroblasts have been done under conditions of serum withdrawal, we also determined the effect of TGF beta on HGF, FGF7, and FGF10 expression in the presence and absence of serum.

## Methods

### Fibroblast isolation and culture

Primary normal fibroblasts were isolated from human lungs from deidentified organ donors whose lungs were not suitable for transplantation. The Committee for the Protection of Human Subjects at National Jewish Health deemed this research as nonhuman subject research. Primary IPF‐derived fibroblasts were isolated from pieces of diseased lung that were resected at University of Colorado Hospital. These specimens are also de‐identified and have been deemed as nonhuman subjects research by the Colorado Multiple Institutional Review Board. The lung was minced and placed on scored tissue culture dishes and cultured with DMEM and 10% FBS. After 1 week fibroblasts migrated out from the minces, and the minces were removed. This culture was expanded up to two times and then frozen down. All subsequent experiments were done with cells passaged less than six additional times.

### Real‐Time RT‐PCR (qPCR)

RNA isolation was done using Qiagen RNeasy Kits, according to the manufacturer's instructions. For real‐time RT‐PCR, the expression levels of genes were expressed as a ratio to the expression of the constitutive probe GAPDH (Wang et al. 2007; Kosmider et al. 2012). The specific verified primers and probes were purchased from Applied Biosystems (Foster City, CA).

### Next generation sequencing of the transcriptome (RNA‐seq)

The RNA‐seq data was generated from cultured fibroblasts from five control subjects (ages 60–80, 4 males and 1 female) and five subjects with IPF (ages 64–69, 3 males and 2 females). Total RNA was isolated using standard kits from Qiagen (Valencia, CA). The isolated total RNA was processed for next‐generation sequencing (NGS) library construction as developed in the NJH Genomics Facility for analysis with a Life Technologies (Carlsbad, CA) Ion Proton NGS platform. A modified Kapa Biosystems (Wilmington, MA) KAPA Stranded mRNA‐Seq kit for whole transcriptome libraries was used to primarily target all poly(A) RNA. Briefly, library construction started from isolation of total RNA species, followed by mRNA (poly[A]) isolation, first and second strand cDNA synthesis, adaptor ligation, amplification, and bead templating. Once validated, the libraries were sequenced as barcoded‐pooled samples on a P1 Ion Proton chip, as routinely performed by the NJH Genomics Facility.

Basecalling and adapter trimming were performed by the Torrent Suite software (version 5.0). Reads of at least 30 nt length were mapped to the canonical chromosomes of the hg19 assembly of the human genome (UCSC) with the STAR RNAseq aligner (version 2.4.1d) (Dobin et al. 2013), using gene annoation data from Ensembl version 75 (http://feb2014.archive.ensembl.org/Homo_sapiens/). The number of reads mapping unambiguously to each gene in the Ensembl annotation was enumerated without strand specificity, using the featureCouts program from the subread package (version 1.5.0‐p1) (Liao et al. 2014). Statistical significance in comparisons was calculated, using the Wald test in the DESeq2 package (version 1.81) (Love et al. 2014) for the R statistical software (version 3.2.0) (R Core Team, R Foundation). We used both pairwise comparison between groups and a model using all of the samples, with the disease status (healthy vs. IPF) and TGF beta (absent vs. present) as categorical variables (the interaction term was not significant and therefore omitted). Principal components analysis (PCA) was performed using the prcomp function in R (version 3.3.2) after normalizing raw count to transcripts per million (TPM). Heat maps were generated, using the pheatmap package (Kolde 2015) in R (version 3.3.2).

### Western blotting

Western blotting was done as described previously in polyacrylamide gradient gels (8–16%; Invitrogen Corporation) run in tris glycine buffer under reduced conditions (Wang et al. 2007). The primary antibodies were smooth muscle actin (A2547; Sigma‐Aldrich, St. Louis, MO) and GAPDH (ab8245; Abcam, Cambridge, MA) The images were quantified, using NIH ImageJ software.

### ELISAs for HGF and FGF7

ELISA analyses were performed using the Human HGF and KGF/FGF7 DuoSet Kit from R&D (DY294; R&D Systems), following the manufacturer's instructions.

### Statistics

Because we were interested primarily in the direction of possible changes, we analyzed changes in mRNA levels using the nonparametric sign test, when the number of samples was six or greater (Conover 1980). If the number of samples was five or fewer the results were evaluated by *t* tests with the assumption that the resulting values were normally distributed. In all analyses, we focused on the magnitude of possible changes in outcomes; this is in keeping with current statistical practices (Curran‐Everett and Benos 2004; Curran‐Everett 2016). Because these analyses were exploratory, we did not control for multiple comparisons. Statistical analyses were done using R 3.3.2 (R Core Team, R Foundation), the SAS/STAT software package, Version 9.3 (SAS Institute Inc. Cary, NC), or GraphPad Prism version 4.0 (GraphPad Software Inc., San Diego, CA).

## Results

To determine the optimal conditions to compare IPF versus control fibroblasts, the effects of TGF beta treatment were first evaluated using control fibroblasts cultured with 5% FBS on tissue culture plastic or within a collagen gel. Fibroblasts grown on tissue culture plastic are under significant tension due to the firm surface, whereas fibroblasts grown within collagen gels should be under minimal tension since the gel can contract. We evaluated the effect of TGF beta on the expression of smooth muscle actin and genes related to the extracellular matrix to confirm previously published findings. Candidate genes were selected because they are well‐known fibrotic genes or are fibroblast derived growth factors that are known mitogens for alveolar type II cells.

**Figure 1:**
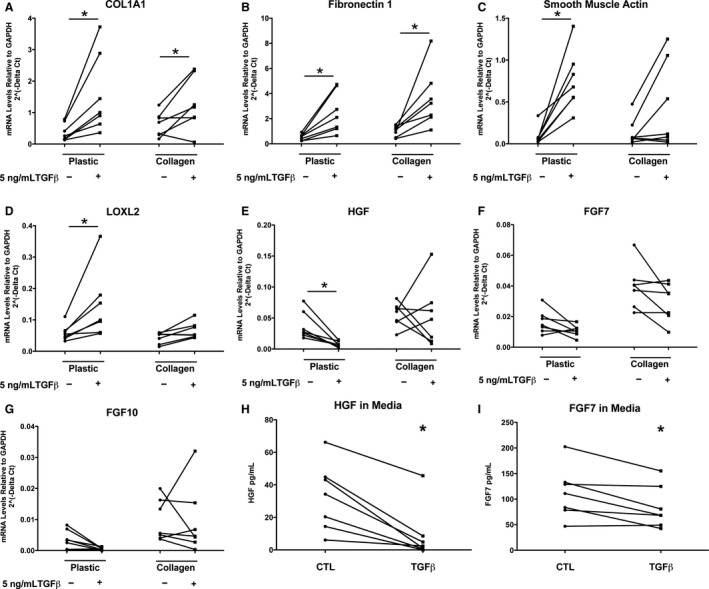
TGF beta 1 increases the expression of extracellular matrix genes and decreases expression and secretion of HGF. Normal lung fibroblasts were plated on tissue culture plastic or within a collagen gel. On day 1 of culture 5 ng/mL of TGF beta 1 was added and the medium was changed to DMEM with 5% FBS. The cells and media were processed 4 days later. The mRNA was quantified by real time quantitative PCR and normalized to the expression of GAPDH. (A) Results for COL1A1; (B) fibronectin; (C) smooth muscle actin; (D) LOXL2, (E) HGF; (F) FGF7 and (G) FGF10, (H) secreted HGF, and (I) secreted FGF7. Fibroblasts from seven different individuals were evaluated for most comparisons, but FGF10 was only measured in six comparisons. *Indicates TGF beta values differ from control values at *P *<* *0.05 by the nonparametric sign test.

TGF beta treatment increased the expression of COL1A1 (collagen type I alpha 1 chain), FN1 (fibronectin), ACTA2 (smooth muscle actin), and LOXL2 (lysyl oxidase like 2) in cells cultured on plastic as expected (Fig. 1). TGF beta also decreased the expression and secretion of HGF in fibroblasts cultured on plastic (Fig. 1). The level of HGF protein secreted in these cultures was at physiologic levels and close to the 50 ng/mL that is routinely used to supplement in vitro cultures (Fig. 1H). TGF beta also decreased FGF7 in the media (Fig. 1I). The basal levels of FGF10 expression were very low in some of the fibroblast isolates and further reduction with TGF beta was difficult to demonstrate. Protein levels of FGF10 were not measured. We also compared the response to TGF beta 2 to TGF beta 1, and the results were similar for these two agonists (data not shown). Overall, the results obtained were much more consistent using fibroblasts cultured on tissue culture plastic than cultured within collagen gels. Thus, for subsequent studies comparing fibroblasts from patients with IPF to control fibroblasts, we chose to culture the cells on tissue culture plastic.

### Comparison of the effect of TGF beta on fibroblasts from IPF subjects and controls

**Figure 2:**
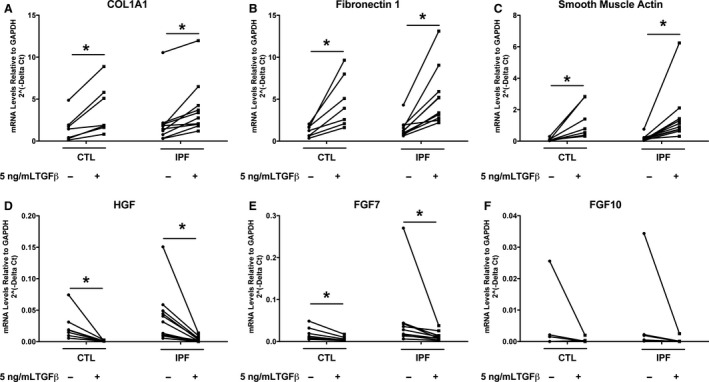
IPF and control fibroblasts respond to TGF beta similarly. Fibroblasts were cultured with 5% FBS with and without 5 ng/mL TGF beta for 4 days as in Figure 1 and the mRNA data quantitated by qPCR. For these experiments we used control fibroblasts from age match donors. There are seven different individual fibroblast isolates for the control and IPF fibroblasts in these experiments for all groups except FGF10 which only has five individual isolates. *Indicates the comparison of the fibroblasts with and without TGF beta by a nonparametric sign test (*P *<* *0.05).

**Figure 3:**
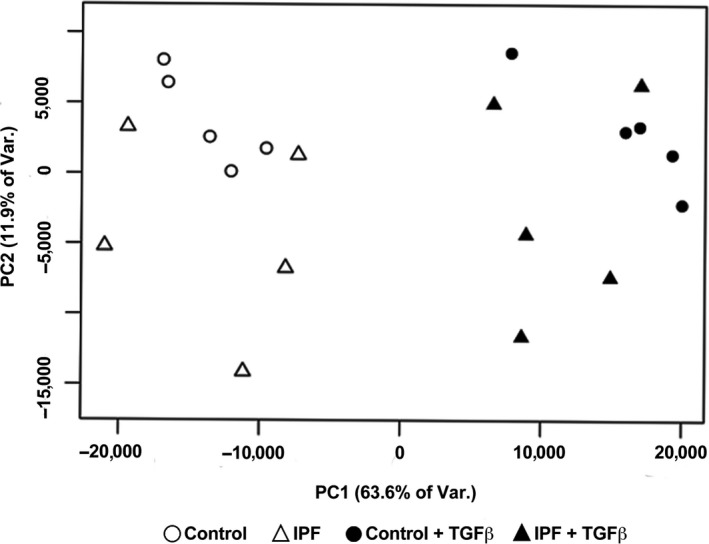
Principal component analysis of gene expression of control and IPF fibroblasts in their response to TGF beta. Samples are plotted according to the first two principal components calculated from normalized expression values (TPM) of nuclear protein‐coding genes as determined by RNA‐seq. Circles correspond to fibroblasts from control subjects, triangles to from fibroblasts from IPF subjects; closed symbols show TGF beta‐treated samples, open symbols untreated controls. There are five individual isolates in each group.

**Figure 4:**
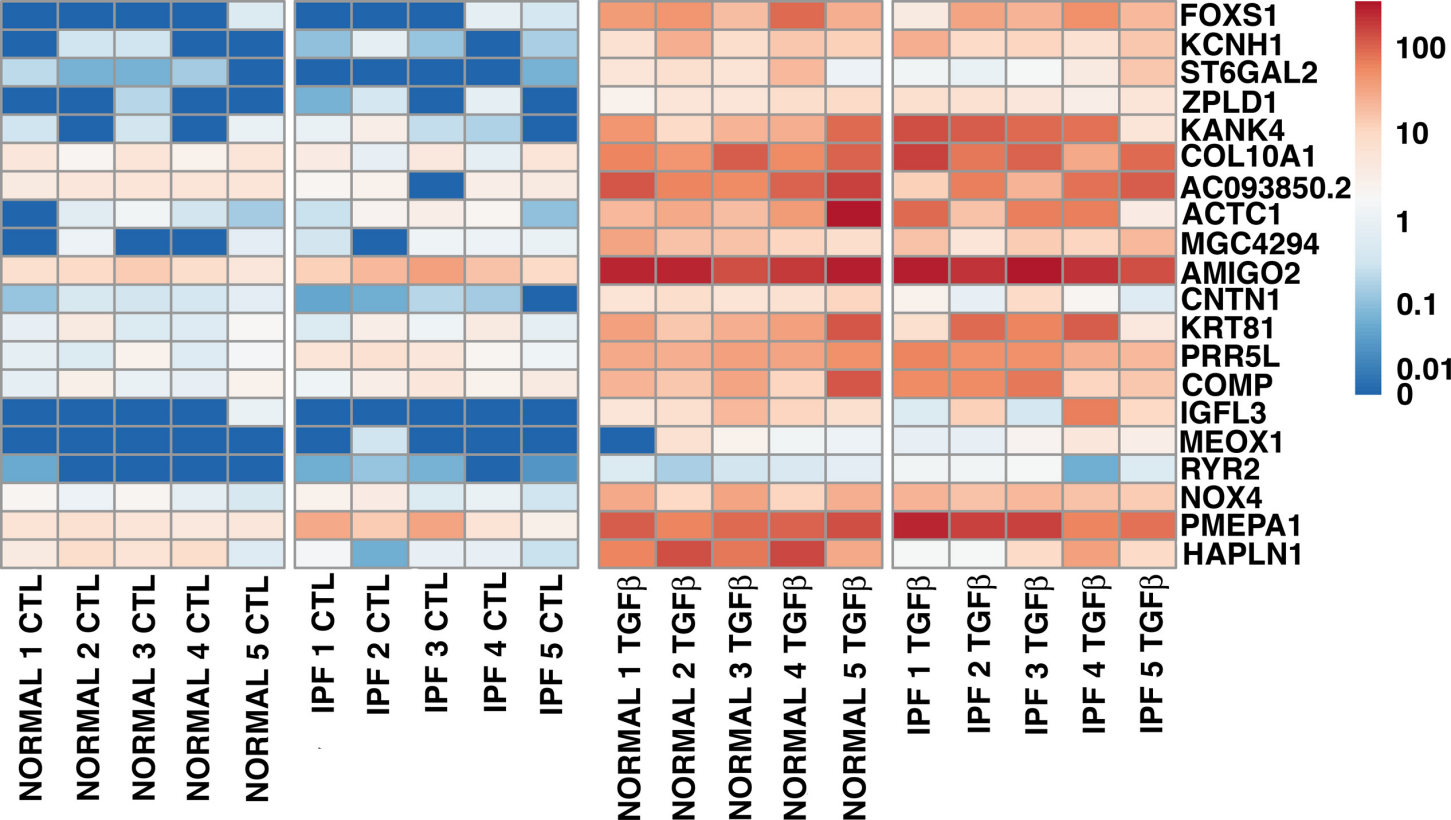
RNA‐seq heatmap of genes up‐regulated by TGF beta. The 20 genes most stimulated by TGF beta from the model using all samples are shown. The fold‐change as computed b DESeq2 was used for sorting, and all adjusted *P*‐values are less than 0.05. Expression values in TPM were transformed before mapping to colors by taking the decadic logarithim after pseudocount of 0.01.

**Figure 5:**
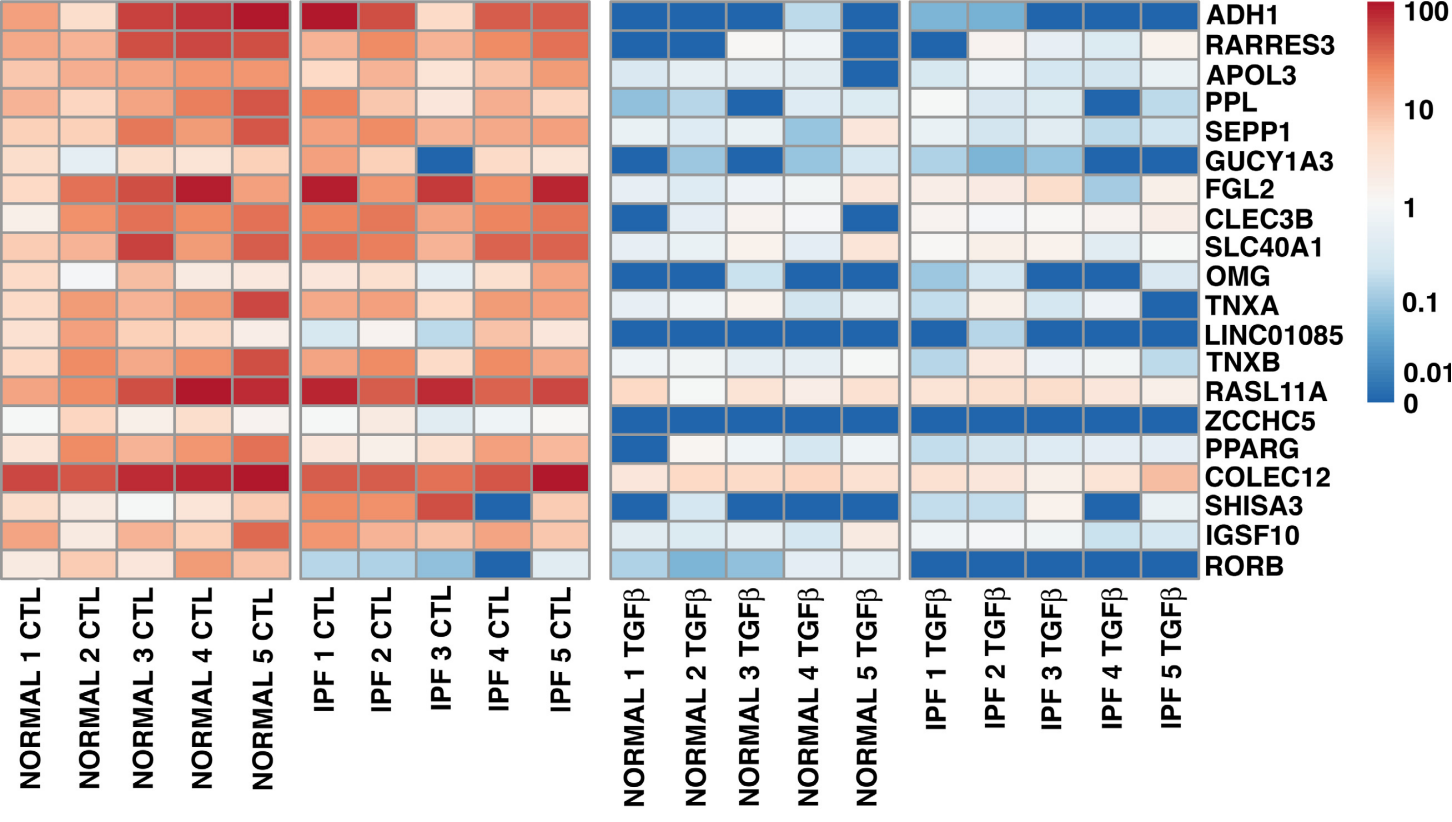
RNA‐seq heatmap of genes down‐regulated by TGF beta. The 20 genes most down regulated by TGF beta are shown. The methods are the same as in Figure 4.

**Figure 6:**
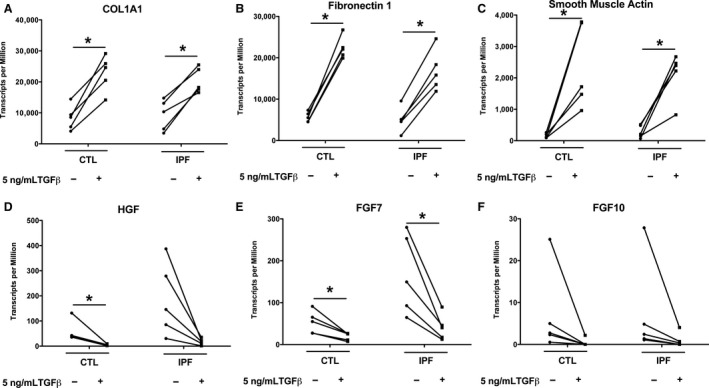
RNA‐seq expression of the response of candidate genes to TGF beta. The normalized expression data as transcripts per million (TPM) for candidate genes as determined by the RNA‐seq analysis. There are five different isolates in each group. The asterisk indicates an adjusted *P*‐value less than 0.05 in the respective pairwise comparison using the Wald test in DESeq2.

In the small gene set that was tested, there was no difference in the basal level of expression or in the response to TGF beta in IPF fibroblasts compared to control fibroblasts cultured on tissue culture plastic in the presence of 5% FBS (Fig. 2). There was a similar increase in expression of COL1A1, FN1, and ACTA2 and inhibition of expression of HGF and FGF7 with TGF beta. To determine more comprehensive differences in mRNA expression between IPF and control fibroblasts under basal and TGF beta‐stimulated conditions we performed a comprehensive genome wide RNAseq analysis. Principal component analysis showed that there was a significant response to TGF beta in both groups of fibroblasts, but in general that response of the IPF fibroblasts was similar to the response of the age‐matched control fibroblasts (Fig. 3). In general, the changes in gene expression after treatment with TGF beta was similar with the control and IPF fibroblasts. The heat maps for the 20 expressed genes most increased by TGF beta and decreased by TGF beta are shown in Figures 4 and 5. In this analysis, the response to the candidate genes was similar to what was shown in Figure 2. In both the control and the IPF fibroblasts, there was a similar increase in COL1A1, FN1, and ACTA2 expression and a decrease in expression of HGF, FGF7, and FGF10 following TGF beta treatment (Fig. 6). The RNAseq data confirms the very low expression of FGF10 compared to the other genes.

**Figure 7:**
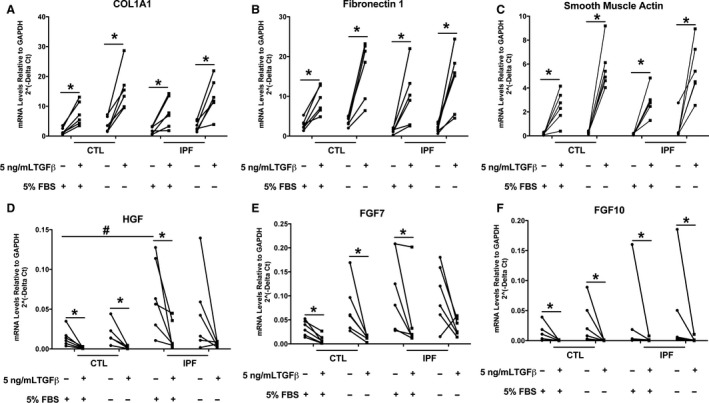
mRNA levels of candidate genes in IPF and control fibroblasts cultured with and without serum. Fibroblasts six control and IPF donors were cultured with 5% FBS or 1 mg/mL BSA in the presence or absence of 5 ng/mL TGF beta for 3 days. The mRNA was isolated and processed as in Figure 1. *Indicates the comparison of the fibroblasts with and without TGF beta by a nonparamentric sign test (*P *<* *0.05).

**Figure 8:**
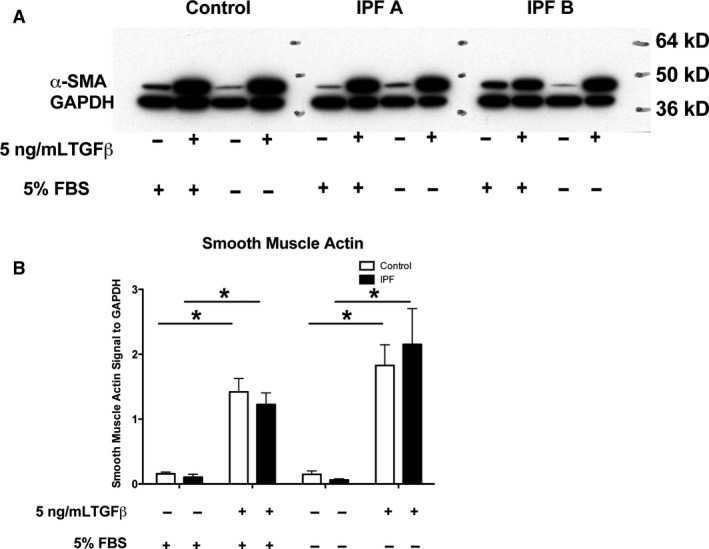
Smooth muscle actin expression in control and IPF fibroblasts. Fibroblasts from control and IPF donors were cultured with 5% FBS or 1 mg/mL BSA in the presence or absence of 5 ng/mL TGF beta for 3 days as in Figure 5. (A) Cell proteins were harvested and quantitated by Western analyses. One control and two IPF samples are shown to indicate the variability in baseline expression. (B). The results from five individuals in each group were quantified by NIH Image‐J software. *Indicates different from control without TGF beta *P *<* *0.05 by a paired t test.

Since we found very little difference between IPF and control fibroblasts when they were cultured with 5% FBS and TGF beta for 4 days and noticed that other investigators cultured their fibroblasts in the absence of serum for 3 days with TGF beta (El Agha et al. 2016), we repeated the studies with our candidate genes and cultured the fibroblasts in the presence and absence of serum for 3 days. TGF beta increased expression of COL1A1, FN1, and ACTA2 in the presence or absence of serum (Fig. 7). Serum withdrawal tended to increase the magnitude of induction of smooth muscle actin and COL1A1 due to TGF beta, but the responses of the control and IPF fibroblasts were similar. In both groups of cells, TGF beta reduced the expression level of HGF, FGF7 and FGF10. In this small series of samples, IPF fibroblasts had higher level of basal expression of HGF in the presence of 5% serum than the control fibroblasts (Fig. 7D). TGF beta increased smooth muscle actin protein expression in the presence or absence of serum similarly in both control and IPF fibroblasts (Fig. 8). The control level of smooth muscle actin tended to be lower in the absence of serum but this was not consistent (IPF A Fig. 8A).

## Discussion

TGF beta is well recognized as an important profibrotic cytokine in the development of pulmonary fibrosis (Khalil et al. 1991). The effect of TGF beta on the regulation of fibroblast matrix genes has been well defined (Sun YB et al. 2016) and was confirmed in this series of experiments. In addition to the stimulatory effects on ACTA2, COL1A1, FN1, and LOXL2, TGF beta greatly suppressed the expression of HGF, FGF7, and FGF10. This is an important finding, because inhibition of growth factor expression would be expected to disrupt normal epithelial‐fibroblast homeostatic interactions. Both HGF and FGF7 lessen alveolar injury, hasten reepithelialization, and prevent fibrosis (Deterding et al. 1997; Dohi et al. 2000; Cahill et al. 2016). HGF is also known as scatter factor and is important in wound healing and the migration of epithelial cells across denuded surfaces. HGF stimulates the closure of scratch wounds by alveolar epithelial cells better than FGF7, insulin‐like growth factor 1, epidermal growth factor, and bone morphogenetic protein 5 (Ito et al. 2015). HGF delivered as a protein or a plasmid and transfected by electroporation prevents bleomycin‐induced pulmonary fibrosis (Dohi et al. 2000; Gazdhar et al. 2013). FGF7 stimulates the differentiation of alveolar type II cells in vitro (Wang et al. 2007) and prevents lung injury due to acid or bleomycin instillation (Yano et al. 1996; Deterding et al. 1997; Sugahara et al. 1998; Volckaert and De Langhe 2014). FGF 10 is expressed predominately in fibroblasts located near type II cells and is thought to be a paracrine factor to maintain the differentiation of these cells (Volckaert and De Langhe 2014; Chao et al. 2016). Mice overexpressing FGF10 are partially protected to the instillation of bleomycin (Gupte et al. 2009). Hence, by decreasing expression of HGF, FGF7, and FGF10, TGF beta would be predicted to inhibit alveolar repair and promote fibrosis. Direct studies on the level of HGF, FGF7, and FGF10 expression in fibroblasts directly isolated and purified from the IPF lung have not been reported. In addition, in situ hybridization studies of growth factor expression in the fibrotic and more normal portions of the IPF lung should be informative.

The effects of TGF beta on fibroblasts are determined in part by the in vitro culture conditions (Arora and McCulloch 1999; Arora et al. 1999). The major determinants are the culture surface and the presence or absence of serum. In determining the effects of TGF beta it is not clear what is the appropriate matrix for culturing the fibroblasts. For the largest and most consistent effects of TGF beta, we used fibroblasts adhering to plastic culture dishes, a very rigid surface. Other investigators have chosen lung extracellular matrix or polymer gels whose rigidity can be altered (Chia et al. 2012; Marinkovic et al. 2013; Parker et al. 2014; Thannickal et al. 2014). Some investigators have suggested that the matrix is a greater determinant of fibroblast function than whether the fibroblasts were isolated from control or IPF lungs (Parker et al. 2014). Fibroblasts cultured within a collagen gel, which should be under minimal tension, had an inconsistent response to TGF beta, especially the response of ACTA2, HGF, and FGF10. The reason for this inconsistency is not known. The effect of TGF beta may require some degree of basal tension. Another important determinant is the presence or absence of serum. Serum removal increases the degree of TGF beta stimulation of smooth muscle actin (Arora and McCulloch 1999). Removal of serum may make the fibroblasts more quiescent and remove some exogenous TGF beta, but it also removes PDGF, insulin like growth factor 1, vibronectin, and other serum factors and may stimulate autophagy. The inhibition of HGF, FGF7, and FGF10 expression by TGF beta was observed with and without serum removal.

The RNAseq data revealed novel genes differentially expressed in TGFβ‐stimulated fibroblasts, whether from control or IPF lungs and confirmed some previous observations. This differential gene expression may provide insight into the pathogenesis of pulmonary fibrosis. During the pathogenesis of pulmonary fibrosis, TGFβ is known to induce the conversion of fibroblasts to myofibroblasts and excessive deposition of extracellular matrix. However, the precise mechanisms by which this occurs are not fully understood. Collagen 10α1, a minor collagen expressed in bone, that has not been studied in pulmonary fibrosis and might be a signal of TGF beta activation. Cartilage oligomeric matrix protein (COMP), a noncollagenous extracellular matrix protein, was highly upregulated by TGFβ as has been reported by others previously but control and IPF fibroblasts responded similarly in our studies (Vuga et al. 2013). Cardiac muscle α actin (ACTC1), KN motif and ankyrin repeat domains 1 (KANK1), and ryanodine receptor 2 (RYR2) are known for their function in actin‐myosin cytoskeletal organization; whether these play a role in myofibroblast differentiation and contraction should be explored. The role of NADPH Oxidase 4 (NOX4), another gene found to be upregulated by TGFβ, in myofibroblast differentiation has been previously established (Hecker et al. 2009; Amara et al. 2010). The most highly upregulated gene, FoxS1, is a transcription factor whose function is poorly understood; its potential role as a master regulator of lung myofibroblast differentiation should be studied. Peroxisome proliferator‐activated receptor gamma (PPARγ) was found to be highly downregulated by TGFβ; its antifibrotic role is well established (Lakatos et al. 2007; Jeon et al. 2014). Periplakin was also highly downregulated may have an antifibrotic function in IPF (Taille et al. 2011). In sum, our RNAseq data provide a resource for investigators aiming to explore the mechanisms promoting fibroblast activation and to identify biomarkers in IPF.

We were surprised to find limited differences in the response to TGF beta between fibroblasts isolated from IPF lungs and lungs from organ donors (Moodley et al. 2003; Hetzel et al. 2005; Vuga et al. 2013). We were not able to demonstrate that the IPF fibroblasts we isolated had higher levels of alpha smooth muscle actin or COL1A1 expression at baseline or in response to TGF beta (Moodley et al. 2003; Roach et al. 2014; El Agha et al. 2016). The general lack of differences between control and IPF fibroblasts could have been for a variety of reasons including the control donor lungs we used, the precise method of explant culture and initial expansion, and the particular in vitro conditions for determining the effect of TGF beta on fibroblasts. All of the lung donors were on a ventilator for several days and received supplemental oxygen. These controls would be expected to be different from controls from lung resections from patients with cancer. We isolated our fibroblasts from outgrowth of explants and then expanded them for a few passages. This is the general method that has been used by other researchers (Larsson et al. 2008; Li et al. 2011; Huang et al. 2014; Parker et al. 2014). However, one of the commonly accepted problems with studying fibroblasts from lungs of IPF patients is that the fibroblasts isolated are likely a mixture of fibroblasts from the normal portions of the lung and the diseased portions of the lung, since the disease process can be patchy. Another confounding factor is that expanding the cells in vitro can alter their in vivo phenotypes.

Although there have been numerous reports comparing fibroblasts from control and IPF lung, we could find no commonly accepted in vitro measurement to establish that the IPF fibroblasts that we isolate and study in our lab are the same phenotype as IPF fibroblasts studied in other labs. To phenotype fibroblasts from IPF patients, researchers will need better surface markers for different subpopulations of fibroblasts and direct dissociation and characterization of the cells at the time of the biopsy or lung transplantation before expansion in vitro. Our results demonstrate that TGF beta decreases expression of HGF, FGF7, and FGF10 in the presence and absence of serum in both control and IPF fibroblasts. Decrease in expression of these growth factors would be expected to impair the ability to repair the alveolar epithelium after injury.
